# Assessment of fatigue in chronic disease: a bibliographic study of fatigue measurement scales

**DOI:** 10.1186/1477-7525-5-12

**Published:** 2007-02-27

**Authors:** Niels Henrik Hjollund, Johan Hviid Andersen, Per Bech

**Affiliations:** 1 Department of Clinical Social Medicine, Institute of Public Health, Aarhus University, Aarhus, Denmark; 2 Center of Public Health, Region Central Jutland, Denmark; 3 Department of Occupational Medicine, Region Hospital Herning, Denmark; 4 Psychiatric Research Unit, Frederiksborg General Hospital, Denmark

## Abstract

A large number of fatigue scales exist and there is no consensus on which fatigue measuring scales that are most appropriate for use in assessment of fatigue in different diseases. We aimed to describe the use of fatigue scales in studies of disease-related fatigue during the last three decades. We searched databases from 1975 to 2004 for original studies reporting on disease-related fatigue and extracted information on method used to assess fatigue, diseases under study and year of publication. A total of 2285 papers reported measures of fatigue in chronic non-acute diseases of which 80% were published during the last decade. We identified 252 different ways to measure fatigue, of which 150 were use only once. Multi-symptom scales (n = 156) were used in 670 studies, while 71 scales specifically designed to measure fatigue were applied in 416 studies. The majority of these studies used scales with a multidimensional approach to fatigue, and most studies used scales that were disease-specific or only applied to few different diseases. Research in disease-related fatigue has increased exponentially during the last three decades, even if we adjust for the general increase in publishing activity. The number of scales has also increased and the majority of scales were developed for specific diseases. There is need for measure instruments with different sizes and dimensionality, and due to ceiling and floor effects, the same scale may not be useful for patients with different severity of fatigue. However, since fatigue is an unspecific symptom there should not be need for adopting disease specific fatigue scales for each individual disease. There may be differences in characteristics of fatigue between diseases and generic measurement instruments may facilitate documentation of such differences, which may be of clinical importance.

## Review

Fatigue may be a clinically important, although subjective and quite unspecific characteristic of some chronic diseases, and major textbooks list diseases from different medical specialities like multiple sclerosis, heart failure, liver disease, adrenal insufficiency, anemia, renal failure, thyrotoxicosis, systemic lupus erythematosus, and any malignant disease [1-3]. Fatigue is also a core symptom in depression [4]. Although we generally know little of etiology of fatigue, we do know that several biological factors exists, e.g. anemia and toxic treatment effects, as well as psychological factors. The word *fatigue *originate from the experience in healthy individuals, but interview based studies have revealed that even though patients label their sensation as fatigue, they often find it qualitatively very different from that fatigue they experienced before they became sick [5]. Such findings indicate that fatigue may not always be sufficiently described as a simple continuum from no fatigue to severe fatigue, and a multidimensional approach has been suggested, including e.g. physical, cognitive, emotional and functional axes [6]. Fatigue may have impact on *quality of life*, and fatigue questions have been included in many *quality of life *scales [6].

Fatigue scales have recently been reviewed elsewhere [6,7]. Dittner et al described and evaluated a number of 30 different scales [6], and concluded that further validation is needed for all scales and that no scale is appropriate for measuring fatigue in all disease groups. Since there is little consensus on which scale possess the most attractive properties, it may be useful to know which scales are actually used to measure fatigue in research of different diseases. The purpose of the present paper is to describe the use of fatigue questionnaires in studies of disease-related fatigue during the last three decades.

## Methods

We searched MEDLINE and PsycINFO for studies reporting on disease-related fatigue in the period 1975 to 2004. The search was last updated February 2004. MEDLINE was searched using the MeSH terms 'fatigue' or 'asthenia', supplemented with a free text search for the terms 'fatigue', 'asthenia' or 'tiredness' occurring together with the MeSH terms 'questionnaires', 'health surveys', 'epidemiologic studies', or 'quality of life'. The PsycINFO database was searched for articles with the descriptor 'fatigue' occurring together with classification codes indicating somatic, functional or psychiatric disease (32xx, 3361, 3363, 337x or 338x). Several check-ups in reference lists suggested that inclusion of other databases would only result in few additional peer-reviewed studies of fatigue in populations of patients.

References without abstract and articles written in non-English language were excluded. All references and abstracts were imported to a database and the following information was extracted from the abstract: method used to assess fatigue, disease(s) under study and year of publication. If it was not possible to extract this information from the abstract, the full-text paper was reviewed. Ad hoc constructed questions and review of medical records were grouped together (ad hoc methods), so was one-item questions used to assess acute treatment side effects. All original studies in adult populations of patients with specified non-acute disease were included in the analyses.

## Results

**Figure 1:**
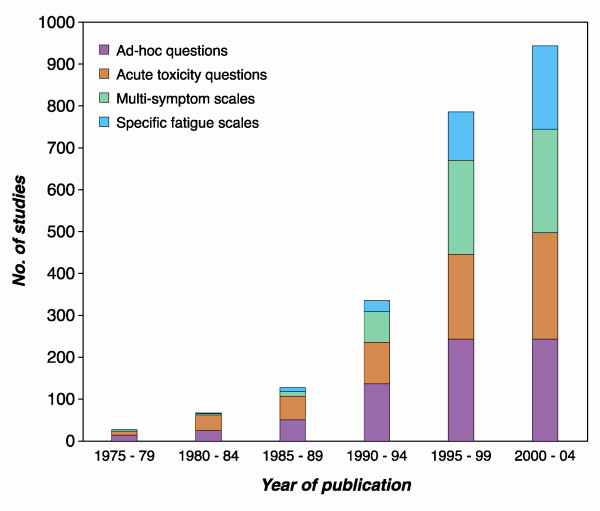
Studies of disease related fatigue by year of publication by method of fatigue assessment.

A total of 2285 papers reporting measures of fatigue in somatic and psychiatric diseases were published between 1975 and 2004. An exponential increase in number of fatigue studies was observed; thus 80% of the studies were published during the last ten years of the period (Fig 1). Ad hoc methods and simple one-item questions to measure acute side effects dominated until 1990 (Fig 1). From the beginning of the 80'ies, *quality of life *scales and other multi-symptom scales were introduced, while scales specifically designed to measure fatigue have mainly been in use the last decade (Fig 1). We identified no less than 252 different methods to assess fatigue of which 150 were used only once. Overall, the most frequent method was ad-hoc constructed questions or retrospective review of medical records, which was used in 669 studies (Table 1).

**Table 1:** Most frequently used questionnaires by type of scale

**Questionnaire**	**Number of studies**
**Ad-hoc methods**	**669**
**Acute side effect assessment question**	**658**
**Multi-symptom scales**	**670**
EORCT QLQ-C30 [9]	131
Short Form-36 [8]	100
Profile of Mood States [10]	88
Chronic Respiratory Disease Questionnaire [11]	20
Sickness Impact Profile [12]	16
Fibromyalgia Impact Questionnaire [13]	11
Symptom Distress Scale [14]	11
Kidney Disease Questionnaire [15]	10
Other multi-symptom questionnaires (n = 148)	283
**Fatigue specific scales**	**416**
Fatigue Severity Scale [16]	68
Fatigue Questionnaire/Fatigue Rating Scale/Chalder Fatigue Scale [17]	48
Multidimensional Fatigue Inventory [18]	35
Piper Fatigue Scale [19]	23
Functional Assessment of Cancer Therapy-Fatigue Scale [20]	22
Fatigue Impact Scale/Fisk Fatigue Severity Score [21]	22
Christensen-Kehlet Ordinal Fatigue Scale [22]	21
Checklist Individual Strength [23]	16
Maastricht Questionnaire (vital exhaustion) [24]	13
Brief Fatigue Inventory [25]	11
Visual Analogue-Fatigue [26]	11
Fatigue Symptom Inventory [27]	10
Multidimensional Assessment of Fatigue [28]	10
Other fatigue-specific questionnaires (n = 58)	106

In 670 studies fatigue was measured by one of 157 multi-symptom scales as one of several domains like the generic questionnaire *Short Form-36 *[8] or the cancer specific *EORTC QLQ-C30 *[9]. In total 71 scales focusing specifically on fatigue was identified in 416 studies (Table 2). Characteristics of the most frequent used fatigue scales are shown in Table 2. The majority of these studies used fatigue scales with a multidimensional approach, typically divided in a physical and a mental part, where the latter may be divided in a cognitive and an emotional part. Some measure instruments also rate functioning (Table 2). Most fatigue scales were developed to measure fatigue in specific diseases, most often cancer, and only few scales have been applied to a wider range of diseases (Table 2).We located 67 studies with a qualitative approach, most often in studies of malignancies (n = 20) and rheumatologic diseases (n = 10).

**Table 2:** Characteristics of the most frequent used specific fatigue scales

**Scale**	**Proposed dimensions**	**Items**	**Time lag**	**Use by decade**	**Most frequent ICD-10 groups (n)**	**Most frequent diseases (n)**
Fatigue Severity Scale [16]	1 (mixed)	9	Not stated	80': 1 90' :26 00': 41	Neurology (33) Rheumatology (12)	Multiple sclerosis (23) SLE (9) Mb Parkinson (7)
Fatigue Questionnaire [17]	Physical fatigue Mental fatigue	11	Not stated	80': 1 90': 15 00': 32	Symptoms (13) Infections (9) Malignancies (9)	CFS (13) Mb Hodgkin (5) HIV (4)
Multidimensional Fatigue Inventory[18]	General fatigue Physical fatigue Reduced activity Reduced motivation Mental fatigue	20	Previous days	90': 14 00': 21	Malignancies (22) Neurologic (4)	Mixed cancer patients (13) Breast cancer (3)
Piper Fatigue Scale [19]	Sensory Affective meaning Cognitive/mood Behavioral/severity	76	Now	90': 13 00': 10	Malignancies (18) Infections (2)	Breast cancer (10) HIV (2)
Functional Assessment of Cancer Therapy – Fatigue [20]	1	13	Past week	90': 2 00': 20	Malignancies (22)	Mixed cancer patients (18)
Fatigue Impact Scale [21]	Cognitive Physical Psychosocial	40	Past month	90': 5 00': 17	Neurologic (13) Infections (4)	Multiple sclerosis (13) Hepatitis C (4)
Christensen-Kehlet [22]	1 (function)	1	Now	80': 8 90': 12 00': 1	Post surgery (20)	Abdominal surgery (20)
Checklist Individual Strength [23]	Experience of fatigue Concentration Motivation Physical activity	20	Past 2 weeks	90': 5 00': 11	Symptoms (6) Malignancies (5)	CFS (6) Multiple sclerosis (2)
Maastricht Questionnaire [24]	1	21	Now	80': 1 90': 7 00': 5	Cardiovascular (12)	AMI (7) Coronar artery sclerosis (5)
Brief Fatigue Inventory [25]	1	9	Now/24 hours	90': 1 00': 10	Malignancies (11)	Mixed cancer patients (7)
Visual Analogue Scale-Fatigue [26]	Energy Fatigue	18	Now	90': 5 00': 6	Malignancies (5)	Mixed cancers (3)
Fatigue Symptom Inventory [27]	Intensity Duration Impact on quality of life	13	Now/past week	90': 5 00': 5	Malignancies (10)	Breast cancer (6) Mixed cancer patients (4)
Multidimensional Assessment of Fatigue [28]	Degree Severity Distress Impact on activities Timing	16	Past week	90': 4 00': 6	Malignancies (5) Rheumatological (3)	Mixed cancers (3)

## Discussion

We located 2285 peer-reviewed papers that reported measures of fatigue in non-acute medical and psychiatric diseases using 252 different ways to measure fatigue. Although we only searched MEDLINE and PsycINFO, we found that inclusion of other databases would only result in few additional peer-reviewed studies of fatigue in populations of patients. We believe our results reflect the way fatigue is assessed in chronic diseases. Two important findings may be highlighted: the large number of studies on disease-related fatigue and the large number of different methods applied to assess disease-related fatigue.

Research in disease-related fatigue has increased exponentially during the last three decades, even if we adjust for the general increase in publishing activity. The true number of studies with fatigue assessments is even higher than reported here, since any application of e.g. SF-36 and even SF-12 involves questions on fatigue, even though results may only be reported as a summary measure that includes contributions from other domains. Also the number of different scales has increased, although the inappropriate uses of homemade ad-hoc questionnaires seem to decline.

The majority of scales were developed for specific diseases. As a consequence it is difficult to assess and analyze differences between different diseases in the occurrence and characteristics of fatigue. Dittner et all argue that the different manifestations and the wide range of mechanisms probably underlying fatigue makes it unlikely that any one fatigue scale will ever be appropriate fore measuring fatigue in all disease groups [6].

It is evident that due to ceiling and floor effects, the same scale may not be useful for both e.g. the terminal cancer patients and the patient with a newly diagnosed multiple sclerosis with subtle symptoms. There may indeed also be need for measure instruments with different sizes and dimensionality. However, since the fatigue symptom is notoriously unspecific, one might question the reason for adopting disease specific fatigue scales for each individual disease. There may be differences in characteristics of fatigue between diseases, although we unfortunately know little about it. Use of generic measurement instruments may facilitate the documentation of such differences, which may be of scientific as well as clinical importance.

## Conclusion

Research in disease-related fatigue has increased rapidly during the last decades. The number of scales has also increased, but the majority of scales were developed for specific diseases. However, since fatigue is an unspecific symptom there is no need for developing disease specific fatigue scales for each individual disease. True enough, there may be differences in characteristics of fatigue between diseases, but only generic fatigue scales may facilitate documentation of such differences.

## Competing interests

The author(s) declare that they have no competing interests.

## Authors' contributions

NHH designed the study, carried out the literature review and data analyses and drafted the manuscript. JHA and PB commented and discussed the analyses and the manuscript. All authors read and approved the final manuscript.
